# Predicting protein stability changes upon mutation using a simple orientational potential

**DOI:** 10.1093/bioinformatics/btad011

**Published:** 2023-01-11

**Authors:** Iván Martín Hernández, Yves Dehouck, Ugo Bastolla, José Ramón López-Blanco, Pablo Chacón

**Affiliations:** Department of Biological Physical Chemistry, Rocasolano Institute of Physical Chemistry, CSIC, 28006 Madrid, Spain; Bioinformatic Unit, Centro de Biología Molecular “Severo Ochoa,” CSIC-UAM Cantoblanco, Madrid 28049, Spain; Bioinformatic Unit, Centro de Biología Molecular “Severo Ochoa,” CSIC-UAM Cantoblanco, Madrid 28049, Spain; Department of Biological Physical Chemistry, Rocasolano Institute of Physical Chemistry, CSIC, 28006 Madrid, Spain; Department of Biological Physical Chemistry, Rocasolano Institute of Physical Chemistry, CSIC, 28006 Madrid, Spain

## Abstract

**Motivation:**

Structure-based stability prediction upon mutation is crucial for protein engineering and design, and for understanding genetic diseases or drug resistance events. For this task, we adopted a simple residue-based orientational potential that considers only three backbone atoms, previously applied in protein modeling. Its application to stability prediction only requires parametrizing 12 amino acid-dependent weights using cross-validation strategies on a curated dataset in which we tried to reduce the mutations that belong to protein–protein or protein–ligand interfaces, extreme conditions and the alanine over-representation.

**Results:**

Our method, called KORPM, accurately predicts mutational effects on an independent benchmark dataset, whether the wild-type or mutated structure is used as starting point. Compared with state-of-the-art methods on this balanced dataset, our approach obtained the lowest root mean square error (RMSE) and the highest correlation between predicted and experimental ΔΔ*G* measures, as well as better receiver operating characteristics and precision-recall curves. Our method is almost anti-symmetric by construction, and it performs thus similarly for the direct and reverse mutations with the corresponding wild-type and mutated structures. Despite the strong limitations of the available experimental mutation data in terms of size, variability, and heterogeneity, we show competitive results with a simple sum of energy terms, which is more efficient and less prone to overfitting.

**Availability and implementation:**

https://github.com/chaconlab/korpm.

**Supplementary information:**

Supplementary data are available at *Bioinformatics* online.

## 1 Introduction

Mutations can affect the stability and function of proteins and eventually cause diseases. Moreover, improving the thermostability of enzymes or antibodies can be crucial for various applications in protein engineering. To study how amino acid substitutions affect protein stability, differences in Gibbs (un)folding free energy (ΔΔG) between the wild-type and mutated protein are routinely estimated by chemical or thermal denaturation experiments. Recent deep mutational scanning methodologies (Fowler and Fields, 2014; Nisthal *et al.*, 2019) are promising high-throughput alternatives but protein stability analysis is typically low throughput. Nevertheless, there is the order of a few thousand ΔΔG stability measures from only a few hundred different proteins with available 3D structure that are collected in databases such as ProTherm (Nikam *et al.*, 2021), ProtaBank (Wang *et al.*, 2018), ThermoMutDB (Xavier *et al.*, 2021) and FireProt (Stourac *et al.*, 2020). Despite its availability and undoubted importance, these data are limited, unbalanced and noisy. Considerable numerical variations can result from differences in experimental conditions and methodologies, such as temperature, pH, concentration, biophysical technique, etc. Mutations to alanine are notably overrepresented from common alanine scanning experiments. The fraction of stabilizing mutations is quite small (less than one-third of the data), but might still be overestimated since many experimental studies seek stabilizing mutations. Taking into account these limitations with variable success (Caldararu *et al.*, 2020; Fang, 2019; Pucci *et al.*, 2018; Sanavia *et al.*, 2020), physics-based (Benedix *et al.*, 2009; Buß *et al.*, 2018; Guerois *et al.*, 2002; Kellogg *et al.*, 2011; Schymkowitz *et al.*, 2005), knowledge-based (Bastolla, 2014; Benedix *et al.*, 2009; Dehouck *et al.*, 2009), sequence-based (Fariselli *et al.*, 2015; Li *et al.*, 2021; Montanucci *et al.*, 2019) and machine learning (Benevenuta *et al.*, 2021; Laimer *et al.*, 2015; Li *et al.*, 2020; Pires *et al.*, 2014; Pires *et al.*, 2014; Quan *et al.*, 2016) computational methods have been developed to predict the effect of mutations on the protein stability. Given the difficulty of the problem, these methods reported a 1.0–2.0 kcal/mol deviation between the predicted and experimental ΔΔG (Pucci *et al.*, 2018). Although current methods have not reached optimal maturity (Fang, 2019; Marabotti *et al.*, 2021; Sanavia *et al.*, 2020), they are useful and costless complementary tools to experimental research.

Instead of tackling the problem by merging a large number of energy terms and/or protein features, we propose here a simpler strategy less prone to overfitting, based on a single knowledge-based potential that only depends on the relative orientation and position of the backbone atoms between residue pairs. This side-chain-independent potential termed KORP, derived from known protein structures by classical Boltzmann inversion, had been successfully used in protein modeling (López-Blanco and Chacón, 2019), and in protein–ligand interactions (Kadukova *et al.*, 2020). Its application to stability prediction required the parameterization of 12 weights to balance the contributions of different amino acid types. This parameterization was done by cross-validation experiments where similarities between training and validation sets were removed. To this end, we built a dataset of ΔΔ*G* mutations clustered by homology, trying to avoid entries that potentially interact with ligands or belong to a protein–protein interface, removing extreme temperature or pH conditions, minimizing the over-representation of alanine and destabilizing mutations.

We evaluated the resulting method, called KORPM, on an independent benchmark dataset that includes both direct and reverse mutations, with the corresponding wild-type and mutated structures (Pucci *et al.*, 2018). Despite its simplicity and speed, KORPM exhibited comparable or better results than state-of-the-art methods. Furthermore, since our approach is almost anti-symmetric by construction, the results were consistent on both direct and reverse mutations. We discuss here these promising results for predicting stability changes upon mutation in the context of the ΔΔ*G* data limitations.

## 2 Materials and methods

The success of KORP is rooted in the consideration of the 6D nature of the residue interactions into a full joint probability distribution as:

(1) $$E_{i.j}^{\mathrm{KORP}}=-\mathrm{RTln}\frac{P^{\mathrm{obs}}\left(r_{ij}.\varphi_{i}.\theta_{i}.\varphi_{j}.\theta_{j}.\omega_{ij}\right)+z}{P^{\mathrm{ref}}\left(r_{ij}.\varphi_{i}.\theta_{i}.\varphi_{j}.\theta_{j}.\omega_{ij}\right)+z},$$

where $P^{\mathrm{obs}}$ is the residue-dependent joint probability of observing a pair of residues *i, j* at a given position and orientation in a non-redundant subset of Protein Data Bank (PDB) structures, and $P^{\mathrm{ref}}$ is the reference probability regardless of residue type. The orientation and position for each interacting pair of residues are described by two polar angles (θ and φ), one torsion angle (ω) and the distance between $C_{\alpha }$ atoms ($r_{ij}$).

Following the same strategy used to successfully adapt this potential to the evaluation of protein–ligand interactions (Kadukova *et al.*, 2020), we balance the contributions of each amino acid type to estimate the impact of a mutation on the unfolding free energy of the protein (Δ*G* = *G*^unfolded^ − *G*^folded^). Here, we define Δ*G* as the unfolding free energy of the protein, and positive values of ΔΔG correspond thus to stabilizing mutations. We obtain a knowledge-based estimation of the wild-type Δ*G*_wt_, and the mutant Δ*G*_mut_ by summing Equation (1) overall native contacts. The change in stability due to the mutation is then expressed as the difference between these two terms:

(2) $$\Delta \Delta G={\Delta G}_{\mathrm{mut}}-{\Delta G}_{\mathrm{wt}}=\sum_{i}^{N}{aa}_{i}{aa}_{\mathrm{mut}}E_{i.\mathrm{mut}}^{\mathrm{KORP}}-\sum_{i}^{N}{aa}_{i}{aa}_{\mathrm{wt}}E_{i.\mathrm{wt}}^{\mathrm{KORP}},$$

where i runs for the residues in contact with the wild-type (wt) or mutated (mut) residue. The weights ${aa}_{i},{aa}_{\mathrm{mut}}$ and ${aa}_{\mathrm{wt}}$ depend on the amino acid type and are fitted to a balanced dataset of experimental ΔΔ*G* values using cross-validation experiments as detailed below.

### 2.1 Data training and validation datasets

We compiled a new curated dataset screening the two largest databases, ThermoMutDB (Xavier *et al.*, 2021) and ProTherm (Nikam *et al.*, 2021), for non-redundant point mutations with known ΔΔ*G* that satisfy the following conditions:

- Experimental temperature between 10 and 40°C and pH between 5 and 9, to avoid extreme conditions. When multiple experiments were available for the same mutation, we selected the one closest to standard conditions (pH 7.0, 25°C), or excluded them if the average difference in ΔΔ*G* exceeds 1.0 kcal/mol. To remove outliers for avoiding distortions of statistical analyses we remove experiments with extreme values (ΔΔG < −7 or ΔΔG > 5.0) kcal/mol.
- Available monomeric structure in the PDB with resolution better or equal to 2.5 Å. The identification of the oligomeric state from the crystal structure and its correspondence with actual ΔΔ*G* experiments are far from optimal. Since protein–protein interactions may affect the free energy estimation, we select only monomeric structures, according to the PDB Biological Assembly, or based on literature information when available.
- No known direct interaction with ligands, since protein–ligand interactions at the mutation site are likely to affect the free energy change. A mutation is discarded if the distance between any pair of heavy atoms in the mutated residue and ligand is smaller than 5 Å.
- We also discard entries with incomplete backbone atoms coordinates and cases outside the two-state folding model.
- Exclude the 684 mutations in the *S*^sym^ blind test dataset (Pucci *et al.*, 2018).

The initial curated dataset (Supplementary Fig. S1) includes 3824 mutations from 139 protein families (sequence identity <25%) with an average ΔΔ*G* of −1.0 kcal/mol and a standard deviation of 1.6 kcal/mol. In total, 73% are destabilizing (ΔΔ*G* < 0), and 27% are stabilizing (ΔΔ*G* > 0), and more than one-third involve alanine. To have a more balanced dataset, we limited the number of entries per pairwise amino acid mutation to a maximum of 15 by removing mainly destabilizing mutations involving alanine. With this data reduction, we aimed at alleviating the alanine over-representation while avoiding losing stabilizing mutations and protein families. The resulting balanced subset (Supplementary Fig. S2) includes 2371 mutations from 129 protein families, 58% destabilizing and 42% stabilizing with an average ΔΔ*G* of −0.7 kcal/mol and a standard deviation of 1.6 kcal/mol. Note that this subset is far from perfectly balanced, e.g. the most frequent amino acid involved in mutations is still alanine (∼20%), while cysteines, tryptophanes and prolines remain underrepresented. Moreover, the majority of the entries belong to a few protein families, i.e. the 10 most populated protein families include around 60% of the total of the mutations.

Despite the relatively small number of different proteins available, low levels of sequence identity between training and test sets are desirable to avoid overfitting and are mandatory for machine learning approaches. This problem complicates fair comparison between methods and can lead to over-optimistic results (Sanavia *et al.*, 2020). To this end, we cluster the mutations in the balanced dataset using MMseq (Hauser *et al.*, 2016) with a sequence identity cutoff of 25% identity. From this clustering, we randomly generated training and validation subsets from distinct clusters to perform our cross-validation experiments as described below.

### 2.2 Reweighing KORP for stability prediction

The original KORP statistical potential (López-Blanco and Chacón, 2019) was extracted from 250 million contacts observed in a non-redundant (<90% identity) dataset of crystallographic structures available in the PDB at the time. KORP was obtained assuming that the frequencies follow a Boltzmann distribution, and it was designed for loop modeling and model quality assessment. As an extra precaution to reduce potential overfitting problems, we repeated here the same procedure as in the original article but excluded the 149 different proteins included in the initial curated dataset and their corresponding homologs.

To apply KORP to stability prediction, we use a reweighing scheme similar to that employed in its adaptation to protein–ligand affinity prediction (Kadukova *et al.*, 2020) (Equation (2)). In this case, we fitted the amino acid depend weighting factors to experimental ΔΔ*G* on a training subset of the balanced dataset and evaluated its performance with a complementary non-redundant validation subset (<50% sequence identity). In the training phase, we minimize mean absolute error (MAE) between predicted and experimental ΔΔG using the BOBYQA optimization algorithm from NLopt (Powell, 2009). In practice, we performed 10- and 5-fold cross-validation, and we repeated each k-fold cross-validation process 100 times to reduce the error in the estimate of mean performance across all protein families. We obtained very similar amino acid weighting factors with *k *=* *5 or 10, with absolute values ranging ∼0.7–2.5 and standard deviations ∼0.1–0.2 (Supplementary Table S1). This low relative variance was also reflected in the RMSE and the Pearson correlation coefficient (PCC) obtained on the validation sets, i.e. 1.38 ± 0.2 kcal/mol and 0.54 ± 0.1, respectively (Supplementary Table S2).

Interestingly, without losing performance, we were able to reduce the number of weighting factors to 12 (Supplementary Tables S1 and S2) by grouping some amino acids of similar chemical types: negative (DE), hydrophobic small (VIL), hydrophobic large (FY), polar uncharged (STNQ) and positive (KH). Since reducing the number of variables is preferable to limit potential overfitting problems, we adopted such reduced weights as a reference. Remarkably, we obtained almost the same weighting factors when mutations in homologous proteins were allowed in the training and validation sets, although with slightly over-optimistic RMSE and PCC values (1.30 ± 0.05 kcal/mol and 0.60 ± 0.04). For cross-checking, we also tested the method on the excluded data points, obtaining worse results, as expected. For example, on the 764 mutations in oligomeric structures or in proximity of ligands, the RMSE is as high as 1.74 kcal/mol and the PCC as low as 0.36 (Supplementary Table S3).

As discussed in Benevenuta and Fariselli (2019) and Sanavia *et al.* (2020), given the average and standard deviation of the dataset and considering an experimental RMSE of 1 kcal/mol, the estimated upper bound of PCC is 0.7. Note that values above these thresholds most likely indicate potential overfitting problems, and the KORPM results are safely below them in all the tested scenarios.

### 2.3 Comparison with other ΔΔ*G* prediction methods

We compare our approach with eight representative ΔΔ*G* predictors based on different strategies. FoldX(v3.0) (Schymkowitz *et al.*, 2005) and EvoFF (Huang *et al.*, 2020) are based on force field potentials, Rosetta Cartesian ddG (CartddG) (Frenz *et al.*, 2020) uses more sophisticated hybrid physical–empirical potentials, DDgun3D (Montanucci *et al.*, 2019) integrates structural information with sequence and evolutionary features, PopMusic-Sym (Pucci *et al.*, 2018) is a symmetric enforced version of the empirical original PopMusic method (Dehouck *et al.*, 2009), and Dynamut2 (Rodrigues *et al.*, 2021), ThermoNet (Li *et al.*, 2020) and ACDCNN (Benevenuta *et al.*, 2021) are machine learning approaches. All predictions were obtained using the same 3D structure, with default method parameters, and assuming standard conditions. Note that FoldX, EvoFF and CartddG required an initial minimization to equilibrate the structures in the corresponding potential. The results of Dynamut2 and PoPMuSiC-Sym were obtained from their respective servers. The ThermoNet predictions were taken from Li *et al.* (2020) and ACDCNN from Pancotti *et al.* (2022). The structures and scripts used are freely available for download (https://github.com/chaconlab/korpm). CartddG, and to a lesser extent FoldX, predicted some destabilization cases with extreme ΔΔ*G* values, likely due to hard collisions. To keep the consistency in the comparison we reduced this anomalous behavior by assigning a value of −8 kcal/mol for all predicted values lower than this cutoff.

### 2.4 Test datasets and performance measures

Two separate datasets were used for performance evaluation and comparison with other stability prediction methods. Firstly, the balanced dataset *S*^sym^ (Pucci *et al.*, 2018), including 342 mutations in 15 protein chains, for which the structure of both the wild-type and mutant protein are available. This dataset includes 342 mutations in 15 protein chains, for which the structure of both the wild-type and mutant protein are available. In addition to those direct mutations (reported in the literature), the dataset also includes all reverse mutations (obtained by anti-symmetry), with a ΔΔ*G* from mutant to wild-type equal but opposite in sign to the direct mutation, which we refer to as anti-symmetry. It contains therefore an equal number of stabilizing and destabilizing mutations. This also permits the characterization of anti-symmetry between direct-reverse mutations for all methods (Fang, 2019; Pucci *et al.*, 2018; Sanavia *et al.*, 2020). Note that a few corrections were made to this *S*^sym^ dataset, after checking the literature (see Supplementary Appendix SI). Secondly, we considered the dataset S669 recently compiled by Pancotti *et al.* (2022). However, we found that it includes a significant number of incorrect entries, as well as many mutations interfering with protein–protein or protein–ligand binding (see details in Supplementary Appendix SII). After the removal or correction of the questionable cases, the remaining mutations were compiled in dataset S461, which was used for comparison of the methods. This curated dataset includes mutations from 44 protein families, 19.1% destabilizing and 80.8% stabilizing, with an average ΔΔ*G* of −1.15 kcal/mol and a standard deviation of 1.26 kcal/mol. For these tests, KORPM was trained with a subset of our balanced dataset, excluding all proteins that present at least 25% sequence identity with any of the proteins from the test set (either *S*^sym^ or S461).

The performances were evaluated using common measures employed in the field, including the PCC the mean absolute error between predicted and the experimental ΔΔ*G* (RMSE), as well as statistical measures of the performance of a binary classification test: sensitivity (TPR), specificity (TNR), precision/recall (PPV), accuracy (ACC) and Matthew’s correlation coefficient (MCC). In addition to single value measures, the receiver operating characteristic (ROC) or precision-recall (PRC) plots, and their area under the curve. PRC is more illustrative of the classifier performance with unbalanced datasets (Saito and Rehmsmeier, 2015). We also adopt an effective and very practical measure described in Frenz *et al.* (2020), based on a three-state classification of errors. They classify mutations as destabilizing if ΔΔ*G* ≤ −1 kcal/mol, stabilizing if the ΔΔ*G* ≥1 kcal/mol, and neutral if it falls between these values. Experimental and predicted mutations are assigned a value of 0 for destabilizing, 1 for neutral and 2 for stabilizing. No difference between these assignations indicates a qualitatively correct prediction, a difference of 1 indicates the prediction was moderately incorrect, (e.g. the mutation is destabilizing but predicted as neutral) and 2 indicates a completely incorrect prediction. Finally, the correlation coefficient between direct and reverse predictions in the *S*^sym^ dataset gives an estimation of the methods’ anti-symmetric bias.

## 3 Results

The prediction scores obtained on the balanced *S*^sym^ dataset are summarized in Table 1. On the complete dataset including both direct and reverse mutations, KORPM obtained an excellent correlation coefficient of 0.69 between predicted and experimental ΔΔG (Table 1, Supplementary Fig. S3), with an RMSE of 1.33 kcal/mol. It only took 3.5 s to predict all the 684 mutations included in the benchmark on a single core of an I7-6770HQ processor. In the subset of direct mutations (Supplementary Table S4), which is more representative of many real-case applications, the RMSE is slightly better, with a value of 1.29 kcal/mol. The somewhat lower PCC value of 0.57 is mainly explained by the smaller range of ΔΔ*G* values (e.g. few direct mutations are highly stabilizing, with ΔΔ*G* > 2 kcal/mol). Overall, these values, which are very similar to those obtained in the cross-validation experiments (Supplementary Table S2), confirm the predictive power of KORPM on this particular dataset.

**Table 1.** Results on independent *S*^sym^ dataset

Methods	RMSE	MAE	PCC	Sc	Of1	Of2	Sen	Spe	PPV	NPV	ACC	MCC	AUC^ROC^	AUC^PRC^	*r* ^Sym^	*δ*
KORPM	**1.33**	**0.95**	**0.69**	**65.9**	**33.7**	0.4	**0.77**	0.79	0.79	**0.78**	**0.78**	**0.56**	**0.86**	**0.86**	0.88	−0.15
Cartddg	3.44	2.63	0.63	52.3	41.1	6.6	0.58	**0.88**	**0.83**	0.68	0.73	0.48	0.81	0.82	0.41	−3.12
FoldX	1.86	1.29	0.54	60.1	34.5	5.4	0.55	0.78	0.71	0.63	0.66	0.33	0.74	0.75	0.27	−1.25
EvoFF	1.56	1.12	0.54	61.7	34.9	3.4	0.61	0.70	0.67	0.64	0.66	0.31	0.74	0.75	0.58	−0.36
PopMusic-S	1.58	1.15	0.52	56.6	42.4	1.0	0.67	0.71	0.70	0.68	0.69	0.38	0.76	0.74	0.77	−0.06
Dynamut2	1.88	1.37	0.38	54.4	38.2	7.4	0.21	**0.88**	0.64	0.53	0.53	0.13	0.62	0.62	0.11	−1.56
DDGun3D	1.43	1.04	0.63	61.8	37.5	0.7	0.68	0.69	0.69	0.69	0.69	0.37	0.75	0.76	**0.99**	−0.04
ThermoNet	1.53	1.09	0.55	58.2	40.9	0.9	0.65	0.70	0.69	0.67	0.68	0.35	0.75	0.74	0.96	−**0.01**
ACDCNN	1.38	1.01	**0.69**	61.5	38.5	**0.0**	0.70	0.79	0.70	0.70	0.70	0.40	0.80	0.80	0.98	−0.05

RMSE, root mean square error; MAE, mean absolute error; PCC, Pearson cross-correlation coefficient; percentages of correct predictions (Sc, same class as experiment), moderately incorrect (Of1, off by one class) or wrong (Of2, off by two classes) according to a three-state classification (destabilizing if ΔΔ*G* ≤ −1 kcal/mol, stabilizing if ΔΔ*G* ≥ 1 kcal/mol, otherwise neutral); Sen, sensitivity; Spe, specificity; PPV, positive predictive value; NPV, negative predictive value; ACC, accuracy; MCC, Matthews correlation coefficient; AUC^ROC^, area under ROC curve; AUC^PRC^, area under PRC curve; *r*^Sym^, absolute value of the correlation of between direct and reverse mutations; *δ*, average sum of direct and reverse mutations. Best results in bold.

**Fig. 1.**
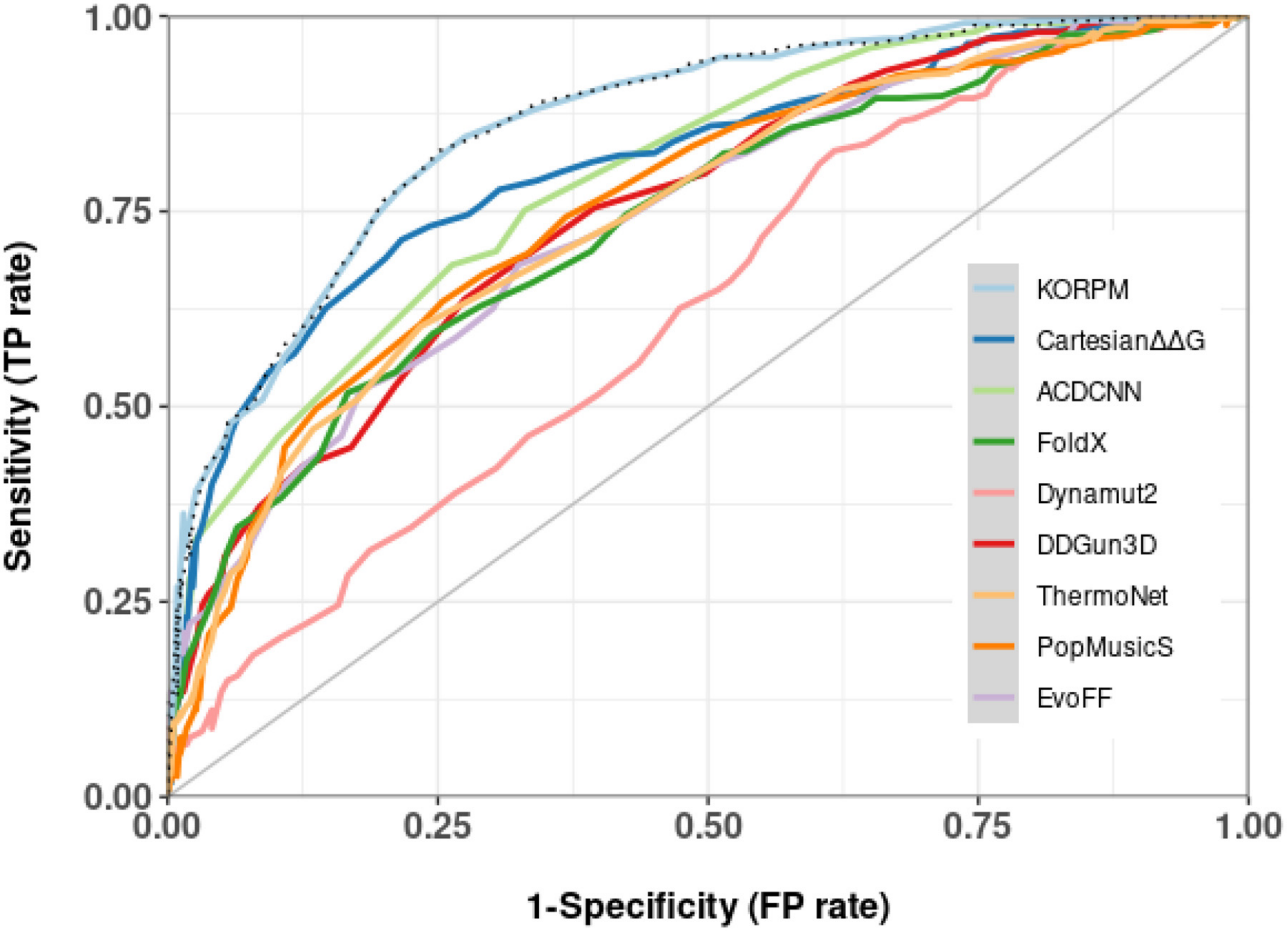
ROC curves obtained by the all the methods on the of the *S*^sym^ dataset. Dashed line corresponds to KORPM results trained excluding all the mutations of *S*^sym^ but including homolog proteins

Binary classification performance metrics such as sensitivity, specificity, PPV, NPV and ACC reported values higher than 0.77 on the complete dataset (Table 1). A more informative indication of the good performances across a wide range of specificity is given by the ROC plot, and the corresponding area under the curve, which is equal to 0.86 (Fig. 1, cyan line). This figure includes two sets of results for KORPM: one where all the mutations of *S*^sym^ were excluded from the training set (dashed line), and another with the stricter criterion of excluding from the training set all proteins presenting at least 25% sequence with any of the *S*^sym^ proteins (blue line). The resulting curves are practically identical, which confirms the absence of significant overfitting with distinct protein families. The high fraction of true positives (i.e. correctly predicted stabilizing mutations) among positive predictions depicted in PRC plot confirms the goodness of the approach (Supplementary Fig. S5A).

If we analyze classification errors in three groups, KORPM identified correctly destabilizing, stabilizing or neutral in 65.9% of the cases, mismatched destabilizing or stabilizing with neutral in 33.7%, and confused destabilizing and stabilizing in only 0.4%.

**Fig. 2.**
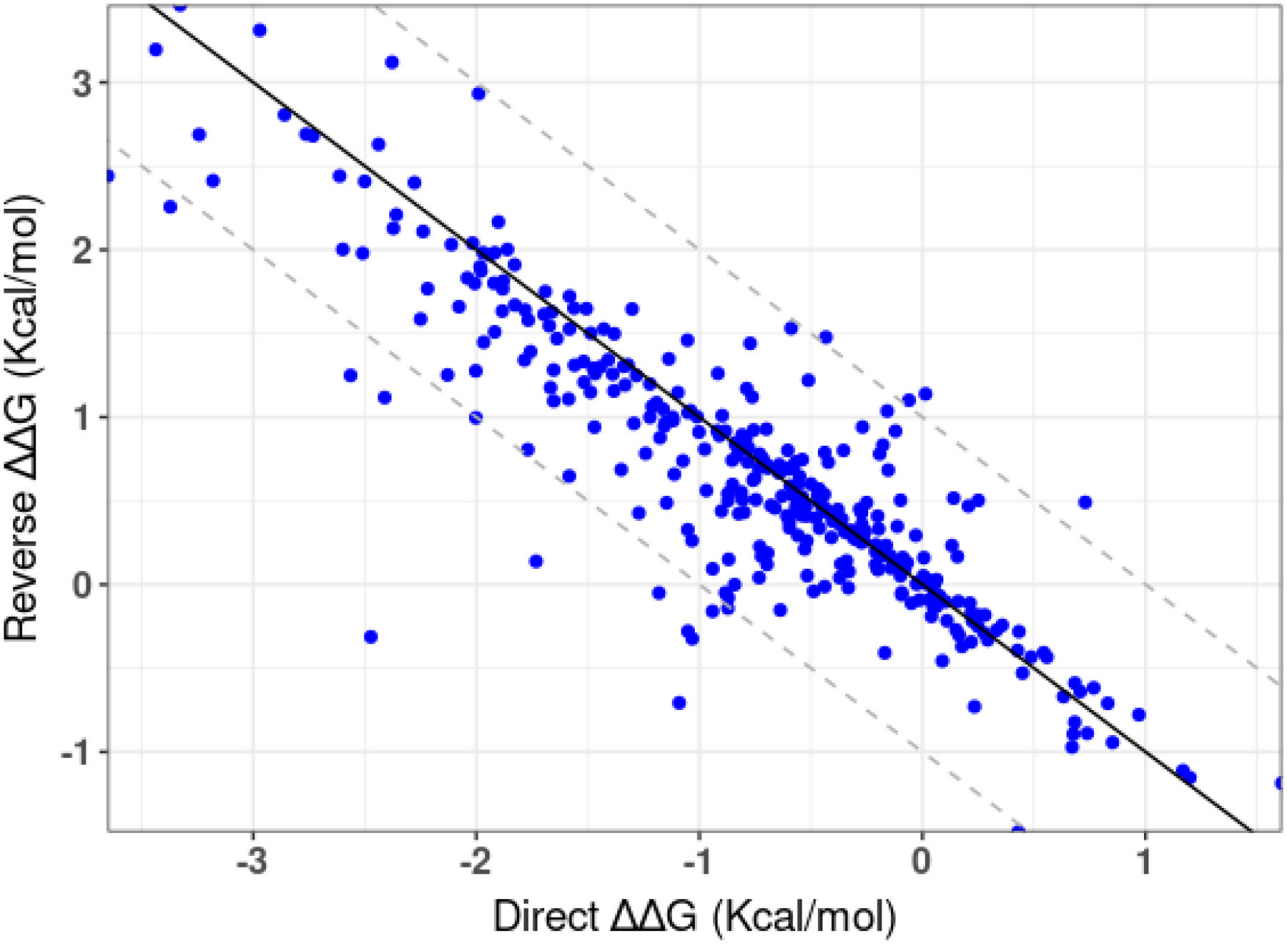
ΔΔ*G* sum of the KORPM predictions for the direct and reverse mutations of the *S*^sym^ dataset. The ideal relationship ΔΔ*G*_direct_ + ΔΔ*G*_reverse_ = 0 is shown as solid line and dashed lines correspond to a variation of ±1 kcal/mol

Our potential is almost anti-symmetric by construction, i.e. the predicted ΔΔG from wild-type to mutant and for the reverse substitution have similar magnitude but opposite sign. This is confirmed by a high correlation of 0.88 between the predicted effect of the direct and the reverse mutation on the *S*^sym^ dataset (Fig. 2). The deviations from anti-symmetry are due to structural differences between the wild-type and the mutant protein, which modify the relative orientations of the mutated residue and its neighbors. Overall, these differences are relatively small, with deviations that are generally <1 kcal/mol.

A detailed investigation of the most prominent outliers, with the largest prediction errors, is interesting as it allows us to identify the limitations of the approach. Flexible regions and the occurrence of conformational changes are obvious sources of imprecision since the free energy is evaluated from a static structure. For example, the mutation Y35G in bovine pancreatic trypsin inhibitor is responsible for a large conformational change from the wild-type (PDB ID: 5PTI) to the mutant (PDB ID: 8PTI) protein (Supplementary Fig. S4). KORPM correctly predicted the sign of the direct mutation but underestimated its magnitude (−3 versus −5 kcal/mol). For the reverse mutation G35Y, the prediction was even worse (−0.4 versus 5 kcal/mol) and corresponds to the data point with the largest error in prediction, and with the largest deviation from anti-symmetry (Fig 2). Large prediction errors are also observed for the two mutations F45A and N43G, which are located also in the flexible loop (Supplementary Fig. S4). On the other hand, the effect on the stability of mutations that occur in binding sites may easily be misjudged by methods that do not explicitly model protein–ligand interactions. For example, the signal transduction protein Chemotaxis Y (PDB ID: 1CEY) has a strongly charged binding site of Mg^2+^ formed by a single Lys and three Asp acids at positions 12, 13 and 57. The suppression of any of them reduces the repellent interaction of the catalytic cleft and stabilizes (2.5–3.0 kcal/mol) the otherwise unstable apo-protein (Sola *et al.*, 2000). Our method fails and predicts a marginal destabilization (from −0.3 to 0.1 kcal/mol). Other sources of error include the fact that protonation states are not included in our potential, and the limited ability to properly detect the formation or destruction of disulfide bridges upon mutation. For example, the V66K mutation in Staphylococcal nuclease (PDB ID: 1EY0) is correctly predicted as destabilizing but with a large error of 5 kcal/mol. This lysine is unusually deprotonated and fully incorporated into the hydrophobic core. The mutation I3C in the lysozyme from bacteriophage T4 (PDB ID: 2LZM) corresponds to an engineered disulfide bridge Cys3–Cys97, and our predictions were wrong by more than 3.5 kcal/mol in both directions.

In summary, despite the expected limitations in handling flexibility, ligand binding, protonation states or SS-bonds, the good overall results confirm the ability and robustness of our approach for the estimation of protein stability.

### 3.1 Comparison with other prediction methods

The comparison between stability prediction methods is difficult because of the different training/testing and validation subsets adopted and the inherent experimental uncertainties. Here, we used the *S*^sym^ dataset for comparison with multiple state-of-the-art methods, even though some of those methods may have included mutations from this dataset in their training process, while we rigorously excluded them. Exceptions are DDGun3D which is an untrained method, and ThermoNet and PoPMusicS which also employed *S*^sym^ as a blind test.

Table 1 shows the comparison with nine ΔΔ*G* predictors on this particular balanced benchmark including both direct and reverse mutations. KORPM obtains the lowest RMSE and MAE, and the highest PCC values. For classification purposes, the ROC and PRC plots and their corresponding area under the curves provide a model-wide evaluation independent of arbitrary threshold definition (Saito and Rehmsmeier, 2015) and demonstrate that our approach has better results at all practical ranges (Fig. 1). At high specificity/low recall, the CartddG protocol also shows good prediction results both in curves and numerically. Overall, CartddG has better specificity, sensitivity and PPV than KORPM. However, CartddG has the poorest RMSE value of all compared predictors, since it tends to predict extreme ΔΔG values for some destabilizing mutations, likely because of hard collisions (even though we tried to reduce this anomalous behavior by capping the predicted values at −8 kcal/mol). If we analyze the three state classification errors devised by CartddG’s authors, the percentage of mutations correctly predicted was ∼66% for KORPM, ∼60–62% for energy-based methods and ∼52% for CartddG while the percentages of totally wrong predictions (destabilizing predicted as stabilizing, or vice versa) is <1% for ACDCNN, KORPM, PopMusicS, DDgun3D and ThermoNet and >5% for CarddgG, Dynamut2 and FoldX.

On the subset of direct mutations, which are more representative of many typical applications of such predictors, Dynamut2 had the lowest RMSE, with KORPM a close second (Supplementary Table S4). Four methods (Cartddg, FoldX, Dynamut2 and ACDCNN) yield PCC values larger than 0.6, while KORPM obtains 0.58. In terms of classification performances, the PPV is particularly important in various real-case applications, since it corresponds to the fraction of mutations that are actually stabilizing among those predicted as stabilizing. All methods yield a lower PPV on the set of direct mutations than on the full set including reverse mutations, but in both cases, KORPM presents very good PPV values (0.62 and 0.82, respectively), second only to Cartddg (0.70 and 0.88, respectively). The ROC/PRC curves (Supplementary Fig. S5B) also indicate that KORPM remains among the top performers in the subset of direct mutations.

If we compare the predictions on direct versus reverse mutations (Table 1, Supplementary Fig. S6), ACDCNN, DDGun3D and ThermoNet achieve almost perfect anti-symmetry with correlation values *r*^Sym^ very close to 1 and with average δ values close to zero (where $\delta ={\Delta \Delta G}_{\mathrm{reverse}}+{\Delta \Delta G}_{\mathrm{direct}}$). With KORPM and PopmusicS, the correlation between direct and reverse mutations is also very high (0.88 and 0.71, respectively), with small δ (−0.15 and −0.06, respectively). These methods achieve generally comparable prediction performances on either direct or reverse mutations, although typically with larger specificity on direct mutants, and larger sensitivity on reverse mutants (Supplementary Tables S4 and S5). On the contrary, the predictions of EvoFF2, CartddG, FoldX and Dynamut2 are significantly better on the direct than on the reverse mutant subsets. This anti-symmetric bias is evidenced by a low correlation between direct and reverse predictions (*r*^Sym^ = 0.58, 0.41, 0.27 and 0.11, respectively). The case of Dynamut2 is particularly striking, as this predictor yields the lowest RMSE on direct mutations (Supplementary Table S4, Supplementary Fig. S5B), but performs extremely poorly on reverse mutations (Supplementary Table S5, Supplementary Fig. S5C). Such an extreme disparity of results might also be due to overfitting, if many of the direct mutations (but not the reverse mutations) were included in training sets of the machine learning methods included in this meta predictor.

Another comparative assessment was performed using the S461 dataset, obtained by curating the S669 recently compiled by Pancotti *et al.* (2022) (see Section 2). The PRC and ROC curves (Supplementary Fig. S7) show top performance for CartddG, closely followed by KORPM and PoPMusicS. Concerning the quality of the ΔΔ*G* predictions (Supplementary Table S7), DDgun3D obtains the best correlation (PCC = 0.63, RMSE = 1.11 kcal/mol) and PopMusicS the lowest RMSE (PCC = 0.61, RMSE = 1.02). ACDCNN also ranks among the top predictors (PCC = 0.61, RMSE = 1.07), followed by KORPM (PCC = 0.57, RMSE = 1.21). As already observed in the *S*^sym^ dataset, CartddG presents a very large error in the predictions (RMSE = 3.59). Overall, these results are rather similar to those obtained on the direct mutations of *S*^sym,^ with the notable exception of FoldX, which performed significantly worse on S461 (PCC = 0.30, RMSE = 1.91). As before, the largest outliers for KORPM correspond to a disulfide bond (0.08 versus −5 kcal/mol, PDB ID 1ITM, mutation C3T) and a salt bridge (−0.18 versus −4.00, PDB ID 2M5S, mutation D303A).

## 4 Discussion

Here, we successfully address the challenge of predicting stability changes upon mutation with a simple sum of pairwise energy terms that depend on the interacting amino acids and their relative position and orientation of three backbone atoms per residue in the native structure. To reduce overfitting, we did not add additional features to our potential, as it is common for several other methods, and we fitted only 12 amino acid-dependent weights using cross-validation procedures. The relatively low variance of those fitted parameters indicates that the method is robust to variations in the training set. We obtained similar performances whether allowing mutations in homologous proteins to be split between the training and validation sets or not, which further suggested the absence of significant overfitting. As a blind test set for comparison with the state-of-the-art methods, we adopted the *S*^sym^ dataset, excluding all of its mutations from the training and cross-validation test. Even though some methods may have incorporated many of such mutations into their training datasets (except for ThermoNet and PoPMusicS which also used *S*^sym^ as a blind test set and DDgun3D which is untrained), KORPM achieved the lowest RMSE and highest PCC between predicted and experimental ΔΔ*G*, as well as superior ROC and PRC curves. In the S461 dataset, our method remains consistently close to optimal with respect to the various performance measures: top ROC curves at 0.19 distance with respect to the best PopMusicS RMSE.

Except for the generally small structural differences between the wild-type and the mutant structures, our approach is almost anti-symmetric by construction, and it performs thus similarly for the direct and reverse mutations. Interestingly, the DDGun3D and ThermoNet methods are almost perfectly anti-symmetric even when there is a large conformational change between the wild-type and the mutant structure. Both methods have good results, which is quite notable since DDGun3D is untrained, and ThermoNet also removed all the *S*^sym^ mutations from the training set. ACDCNN has top results on reverse mutations while maintaining good results on direct cases including the S461 dataset. In contrast, methods like Cartddg, FoldX and Dynamut2 perform very well on direct mutations, which may be more relevant for some types of real-case applications, but generally worse on reverse mutations, as has been previously noted (Fang, 2019; Pucci *et al.*, 2018; Sanavia *et al.*, 2020). Dynamut2 is a meta-predictor that includes several machine learning-based algorithms with known overfitting problems (Fang, 2019) that may contribute to the drastic differences in performances between direct and reverse mutations. CartddG and FoldX appear sensitive to structural variations, reflected by the higher dispersion of their predicted ΔΔ*G* values. One of the major limitations of CartddG is that around 10% of the test cases yield exceptionally high destabilizing values, compromising quantitative predictions of ΔΔ*G*. Nevertheless, this method has top ROC and recall curves, particularly with direct mutations with both *S*^sym^ and S461 datasets. Whether it is preferable to perform well on direct mutations, or to respect the anti-symmetry, may depend strongly on the type of application. But either way, the clear advantage of KORPM is that it stands among the top predictors for direct mutations, while still maintaining a very high level of anti-symmetry and speed.

By taking a close look at KORPM outliers, we identified some expected limitations which are also common to most approaches for predicting ΔΔ*G*: the poor handling of conformational changes upon mutation (our method, as most others, completely neglects them, while some methods like FoldX or Cartddg include a short minimization), the difficulty to account for changes in protonation states or disulfide bonding, as well as the interferences from protein–ligand or protein–protein interactions.

We showed that KORPM has comparable or better performances than competing methods but such comparisons between methods should be always considered with a certain degree of caution. Besides the difficulty to identify overfitted methods without purely novel and non-redundant data, there are still many sources of uncertainty in the available experimental ΔΔ*G* data itself. Firstly, the most critical point is that the amount of data is limited, with no more than a few thousand mutations in a few hundred distinct proteins. The variability in terms of protein folds, functions or amino acid nature is also rather small, and no more than a third of currently available mutations are stabilizing. Furthermore, the data are far from homogeneous as it includes measurements made with different experimental methods, and in different environmental conditions (temperature, pH, additives, etc.), which creates a numerical variability that can go way beyond the experimental errors. To definitively improve and properly cross-validate protein stability prediction methods, it would be mandatory to increase the amount of mutational data by orders of magnitude.

Secondly, there is no genuine and perfect correspondence between the available crystallographic structure and the soluble structure in the conditions of the experiment. Potential changes of the oligomeric state, presence or absence of a particular ligand, conformational changes or partial aggregation are critical but largely ignored intrinsic limitations of ΔΔ*G* predictions. Notably, if the mutation is located at a protein–protein interface or protein–ligand binding site, it becomes a different prediction problem, related to the binding affinity. And of course, since experimental research is typically focused on the protein’s biological activity, functional and interaction sites are often chosen as targets for mutagenesis. Even if the ΔΔ*G* measurements are explicitly performed on the apo form, active sites tend to have properties distinct from the rest of the protein, with significantly more options for stabilizing the fold via mutations, but often at the cost of functionality (Dehouck *et al.*, 2011; Jacquier *et al.*, 2013). For example, eight state-of-the-art prediction methods reported a weak correlation between predicted and experimental ΔΔ*G* for 51 mutants of β-glucosidase around its binding site (Huang *et al.*, 2020). Moreover, in the most common protein design application of maintaining stable and active a given target protein at higher temperatures, one has to search for thermal stabilizing mutations without affecting the function of the protein, and thus binding regions should be excluded. We tried to alleviate these issues by removing from the training and validation sets any mutations near ligand binding sites or protein–protein interfaces. However, precise information about the presence of specific ions or ligands and the correct oligomeric state of the mutated and wild-type proteins is often unavailable in the specific conditions of the ΔΔ*G* measurement. Note also that the experimental characterization of the oligomerization state and the correspondence of the PDB structure is totally ignored in the current databases.

Thirdly, the very definition of the target quantity, the stability change upon mutation, is not without ambiguity. The scope of most predictors is limited to a simple two-state folding model. Any deviations from this model, or occurrences of higher-order transitions with multiple intermediates, are bound to generate inaccuracies, and this information is rarely well translated into database annotations. Furthermore, most predictors are essentially blind to the different types of contributions that participate in the change in free energy. Notably, entropic contributions are critical, but very rarely taken explicitly into consideration. Some methods based on effective energy functions may implicitly include solvent entropy but do not account for changes in the conformational entropy of the protein. On the other hand, ΔΔ*G* is the change of the free energy difference between the native and non-native or unfolded state of a protein, but the effect of the mutation on the free energy of the non-native state is usually neglected. As an exception, the DDGREM contact potential (Bastolla, 2014), which is based on the same contact potential included in DDgun3D, is also able to estimate the free energy of the non-native state through a Random Energy Model. However, this potential was calibrated with a limited number of known structures and tested only on a small subset of mutations. We plan to improve and test it more specifically in the future.

Despite this realistic view of the limitations of the quality and availability of data, and in consequence of our ability to properly assess predictive methods, there is room for future improvements. The higher throughput afforded by new deep mutational scanning methodologies (Nisthal *et al.*, 2019) has the potential to revolutionize the field by adding large-scale mutational data of specific proteins. Freely available AlphaFold structure predictions (Jumper *et al.*, 2021), and in particular, high confidence models can also have an impact on rescuing ΔΔ*G* experimental data orphans of 3D protein structures.

In summary, we believe that our approach constitutes a very valuable addition to the field, thanks to its simplicity, its natural robustness against overfitting, and its good balance between efficiency and accuracy. These characteristics make it likely that further advances in prediction performances could be achieved by combining/integrating it with other methods, or with some of the energetic functions or features considered by those methods. Future work should address merging our structural base potential with complementary evolutionary information derived from multiple sequences that have been proven useful to predict mutations. Other weaknesses of the proposed method such as protonation and disulfide bonding could be alleviated with ad hoc modules for adding effective correction factors. Meanwhile, KORPM offers a costless alternative to estimate protein stability changes upon mutation, with an accuracy comparable to or better than currently available methods.

## Supplementary Material

btad011_Supplementary_Data
