# Supplementary material for: Predicting protein stability changes upon mutation using a simple orientational potential
Source: Bioinformatics. 2023 Jan 11;39(1):btad011. doi: 10.1093/bioinformatics/btad011 (PMC9850275; doi:10.1093/bioinformatics/btad011)
Supplement: btad011_Supplementary_Data [file btad011_supplementary_data.zip › KORPM_SI_R1 final.pdf]

Supplementary Information for

Predicting protein stability changes upon mutation using a simple orientational potential

Iván Martín Hernández[1], Yves Dehouck[2], Ugo Bastolla[2], José Ramón López-Blanco[1], Pablo Chacón[1*].

TABLE OF CONTENTS

Table S1. KORPM cross-validation weights

Table S2. KORPM cross-validation results

Table S3. KORPM results with removed cases.

Table S4. Results on direct mutations of S^{sym} dataset

Table S5. Results on reverse mutations of S^{sym} dataset**.**

Figure S1. Initial curated dataset.

Figure S2. Balanced dataset.

Figure S3. ΔΔG estimations obtained by nine methods tested on S^{sym} dataset.

Figure S4. Outlier mutations bovine pancreatic trypsin inhibitor.

Figure S5. Comparative Receiver Operating Characteristic (ROC) or Precision-Recall (PRC) curves on S^{sym} dataset.

Figure S6. Comparative ΔΔG anti-symmetry results.

Figure S7. Comparative Receiver Operating Characteristic (ROC) or Precision-Recall (PRC) curves on S461 dataset.

Table S7. Results on direct mutations of S461 dataset (curated from S669).

Appendix I. Corrections of original S^{sym} dataset.

Appendix II. Corrections of S669 dataset.

Ssym.xlsx. Excel file with the results obtained with S^{sym} dataset.

S461.xlsx. Excel file with the results obtained with S461 dataset.

Table S1. KORPM cross-validation weights

aa_i	K-fold	A	C	D	E	F	G	H	I	K	L
20	5	1.89±0.10	0.73±0.11	0.86±0.09	0.93±0.12	2.37±0.11	0.99±0.21	1.13±0.08	2.50±0.12	0.99±0.12	2.62±0.16
20	10	1.88±0.07	0.73±0.09	0.87±0.07	0.92±0.07	2.37±0.09	0.97±0.13	1.13±0.04	2.51±0.09	0.99±0.09	2.63±0.12
12	5	1.88±0.14	0.72±0.11	0.88±0.08	0.88±0.08	2.44±0.11	0.97±0.25	1.03±0.09	2.51±0.15	1.03±0.09	2.52±0.14
12	10	1.87±0.10	0.73±0.08	0.87±0.05	0.87±0.05	2.45±0.08	0.97±0.18	1.03±0.06	2.52±0.11	1.03±0.06	2.52±0.10
		**M**	**N**	**P**	**Q**	**R**	**S**	**T**	**V**	**W**	**Y**
20	5	1.66±0.22	1.22±0.08	1.16±0.15	1.00±0.05	1.62±0.13	1.37±0.11	1.16±0.14	2.42±0.19	1.50±0.10	2.55±0.18
20	10	1.67±0.16	1.23±0.06	1.16±0.09	0.99±0.04	1.61±0.11	1.36±0.08	1.16±0.16	2.43±0.14	1.50±0.08	2.57±0.13
12	5	1.65±0.22	1.11±0.07	1.42±0.08	1.11±0.07	1.61±0.13	1.11±0.07	1.12±0.07	2.51±0.14	1.42±0.08	2.44±0.11
12	10	1.66±0.16	1.11±0.05	1.40±0.05	1.11±0.05	1.60±0.10	1.11±0.05	1.12±0.05	2.53±0.10	1.40±0.05	2.45±0.08

The aa_i amino acid type weights factors are either 20 that corresponds to the canonical types or 12 that correspond to a reduction into the following groups negative (DE), hydrophobic small (VIL), hydrophobic large (FY), polar uncharged (STNQ), and positive (KH). 5 and 10-fold cross-validation were performed. The standard deviations correspond to 100 independent k-fold cross-validation runs where similarities between training and validation sets were removed.

Table S2. KORPM cross-validation results

aa_i	K-fold	RMSE	MAE	PCC	Sen	Spe	PPV	ACC	MCC	AUC^{ROC}	AUC^{PRC}
20	5	1.38±0.27	1.03±0.19	0.54±0.07	0.51±0.07	0.83±0.03	0.65±0.09	0.71±0.06	0.36±0.08	0.77±0.04	0.64±0.09
20	10	1.38±0.29	1.03±0.22	0.52±0.09	0.52±0.09	0.83±0.05	0.65±0.13	0.72±0.07	0.37±0.11	0.78±0.05	0.64±0.13
12	5	1.38±0.27	1.03±0.20	0.55±0.07	0.53±0.07	0.84±0.02	0.67±0.09	0.72±0.06	0.39±0.07	0.77±0.04	0.65±0.09
12	10	1.38±0.28	1.02±0.21	0.53±0.09	0.54±0.09	0.84±0.04	0.66±0.12	0.73±0.06	0.39±0.10	0.78±0.05	0.64±0.13

RMSE Root Mean Square Error; MAE Mean Absolute Error; PCC Pearson Cross-correlation Coefficient; Sen Sensitivity, Spe Specificity; PPV positive predictive value; NPV negative predictive value; ACC accuracy; MCC Matthews correlation coefficient; AUC^{ROC} Area under ROC curve; AUC^{PRC} area under PRC curve

Table S3. KORPM results with removed cases.

Mutation Dataset	#Total	#stabilizing	#destabilizing	RMSE	MAE	PCC	Sen	Spe	PPV	MCC
Monomers with close ligand mutations	306	70	236	1.88	1.40	0.21	0.23	0.86	0.33	0.10
Oligomers	773	193	580	1.72	1.28	0.37	0.36	0.81	0.40	0.22
Oligomers without close ligand mutations	687	158	529	1.72	1.27	0.37	0.39	0.82	0.39	0.21
Initial curated dataset	3824	1061	2763	1.42	1.03	0.57	0.52	0.80	0.50	0.32
†Balanced subset	2371	993	1378	1.38	1.03	0.55	0.53	0.84	0.67	0.39

†Data taken from Table S2 using the reference values of AA=12 and K-fold 5.

Table S4. Results on direct mutations of S^{sym} dataset.

METHOD	RMSE	MAE	PCC	Sc	Of1	Of2	Sen	Spe	PPV	NPV	ACC	MCC	AUC^{ROC}	AUC^{PRC}
**KORPM**	1.28	0.92	0.57	67.8	31.6	0.6	0.42	0.91	0.62	0.82	0.78	0.37	0.81	0.55
**Cartddg**	3.32	2.66	**0.68**	57.9	40.4	1.7	0.56	0.92	**0.70**	0.86	**0.83**	**0.52**	**0.84**	**0.64**
**FoldX**	1.48	1.06	0.65	**68.1**	**30.1**	1.8	**0.72**	0.76	0.52	**0.89**	0.75	0.44	0.80	0.59
**EvoFF**	1.36	1.00	0.57	66.4	32.2	1.4	0.62	0.75	0.47	0.85	0.72	0.34	0.76	0.50
**PopMusic-S**	1.56	1.14	0.47	57.6	41.2	1.2	0.62	0.72	0.44	0.84	0.70	0.31	0.75	0.44
**Dynamut**	**1.20**	**0.89**	0.63	67.2	31.9	0.9	0.32	**0.93**	0.60	0.79	0.77	0.31	0.80	0.55
**DDGun3D**	1.42	1.03	0.55	62.9	36.2	0.9	0.52	0.75	0.42	0.82	0.69	0.25	0.71	0.40
**ThermoNet**	1.52	1.09	0.47	58.2	40.9	0.9	0.60	0.72	0.43	0.83	0.69	0.29	0.71	0.41
**ACDCNN**	1.35	0.99	0.61	62.4	37.4	**0.0**	0.44	0.79	0.43	0.80	0.70	0.23	0.70	0.45

RMSE Root Mean Square Error; MAE Mean Absolute Error; PCC Pearson Cross-correlation Coefficient; Percentages of correct predictions (Sc, same class as experiment), moderately incorrect (Of1, off by one class), or wrong (Of2, off by two classes) according to a three-state classification (destabilizing if $\Delta\Delta G \leq -1$ kcal/mol, stabilizing if $\Delta\Delta G \geq 1$ kcal/mol, otherwise neutral); Sen Sensitivity, Spe Specificity; PPV positive predictive value; NPV negative predictive value; ACC accuracy; MCC Matthews correlation coefficient; AUC^{ROC} Area under ROC curve; AUC^{PRC} Area under PRC curve.

Table S5. Results on reverse mutations of S^{sym} dataset**.**

METHOD	RMSE	MAE	PCC	Sc	Of1	Of2	Sen	Spe	PPV	NPV	ACC	MCC	AUC^{ROC}	AUC^{PRC}
**KORPM**	**1.38**	**0.98**	0.49	**64.0**	35.7	0.3	0.90	0.45	0.82	**0.61**	**0.78**	**0.39**	**0.80**	**0.92**
**Cartddg**	3.55	2.60	0.45	46.8	41.8	11.4	0.59	0.76	0.88	0.39	0.63	0.31	0.74	0.89
**FoldX**	2.18	1.51	0.38	52.0	38.9	9.1	0.49	**0.82**	**0.89**	0.36	0.58	0.28	0.69	0.88
**EvoFF**	1.75	1.26	0.31	57.0	**37.7**	5.3	0.61	0.57	0.80	0.34	0.60	0.15	0.65	0.85
**PopMusic-S**	1.60	1.16	0.47	55.6	43.5	0.9	0.69	0.66	0.85	0.43	0.69	0.31	0.75	0.90
**Dynamut2**	2.38	1.85	0.05	41.6	44.4	14.0	0.17	0.75	0.67	0.24	0.33	-0.08	0.44	0.72
**DDGun3D**	1.45	1.05	0.53	60.8	38.6	0.6	0.74	0.53	0.82	0.42	0.69	0.25	0.70	0.88
**ThermoNet**	1.53	1.10	0.47	57.6	41.5	0.9	0.67	0.64	0.84	0.41	0.67	0.28	0.72	0.88
**ACDCNN**	1.41	1.03	**0.58**	60.5	39.5	**0.0**	0.79	0.44	0.80	0.43	0.70	0.23	0.75	0.90

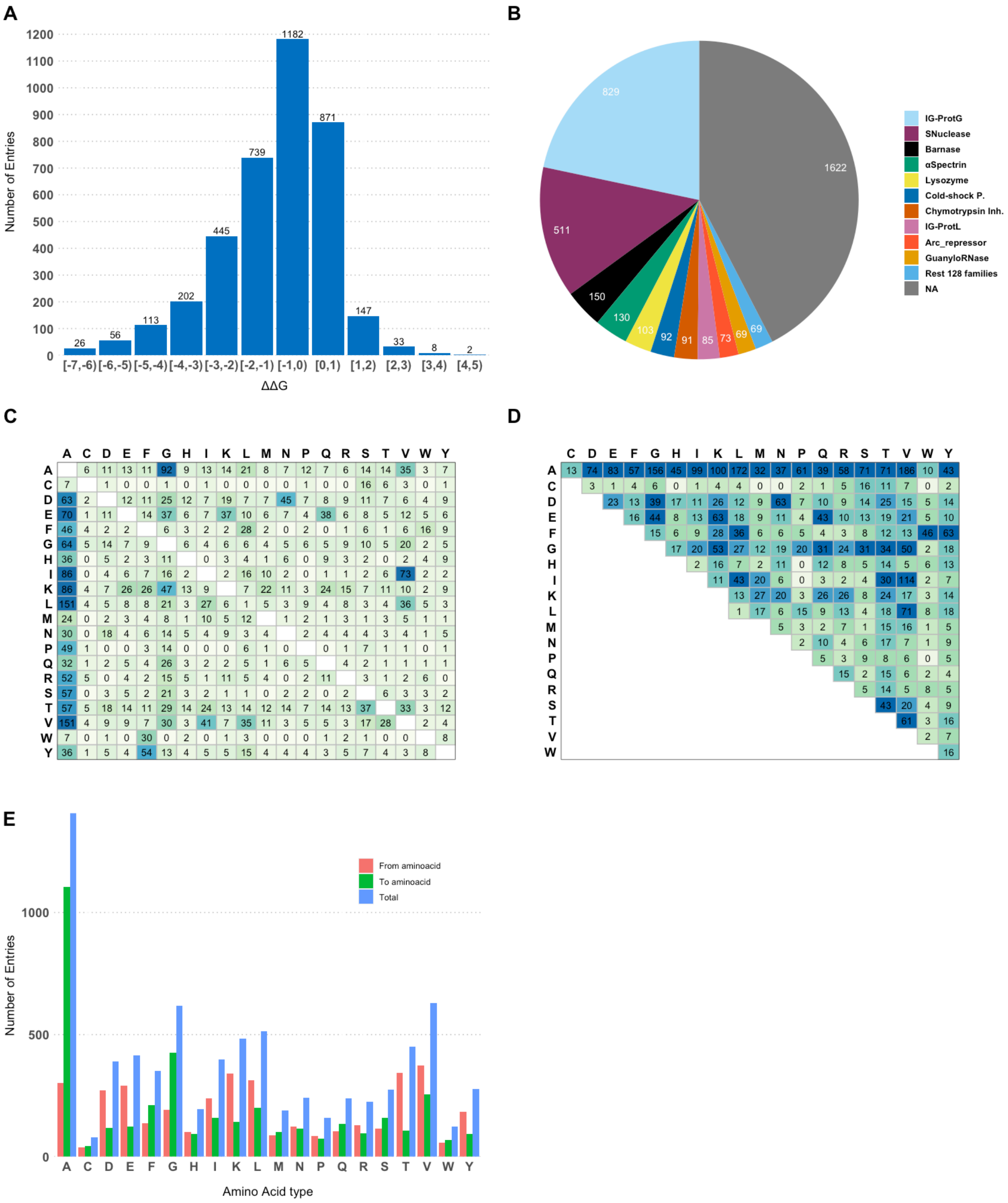

Figure S1. Initial curated dataset. We compiled a new curated dataset screening the two largest databases, ThermoMutDB (Xavier, et al., 2021) and ProTherm (Nikam, et al., 2021), for non-redundant point mutations with know ΔΔG trying to avoid entries that potentially interact with ligands or belong to a protein-protein interface and removing extreme temperature or pH conditions (see details on the main text). This initial curated dataset includes 3824 mutations from 139 protein families with an average ΔΔG of -1.0 kcal/mol and a standard deviation of 1.6 kcal/mol. In total, 72% are destabilizing (ΔΔG<0) and 28% are stabilizing (ΔΔG>0). Panel A) Distribution of the number of mutation entries with respect to ΔΔG values. Panel B) Protein families included in the dataset (sequence identity <25%). Panel C) Number of mutations for a given pair-wise substitution. Panel D) Sum of the pair-wise substitutions of panel C. Panel E) Number of mutations of a given amino acid type to another (orange box) and the number of mutations to a given amino acid type (green box).

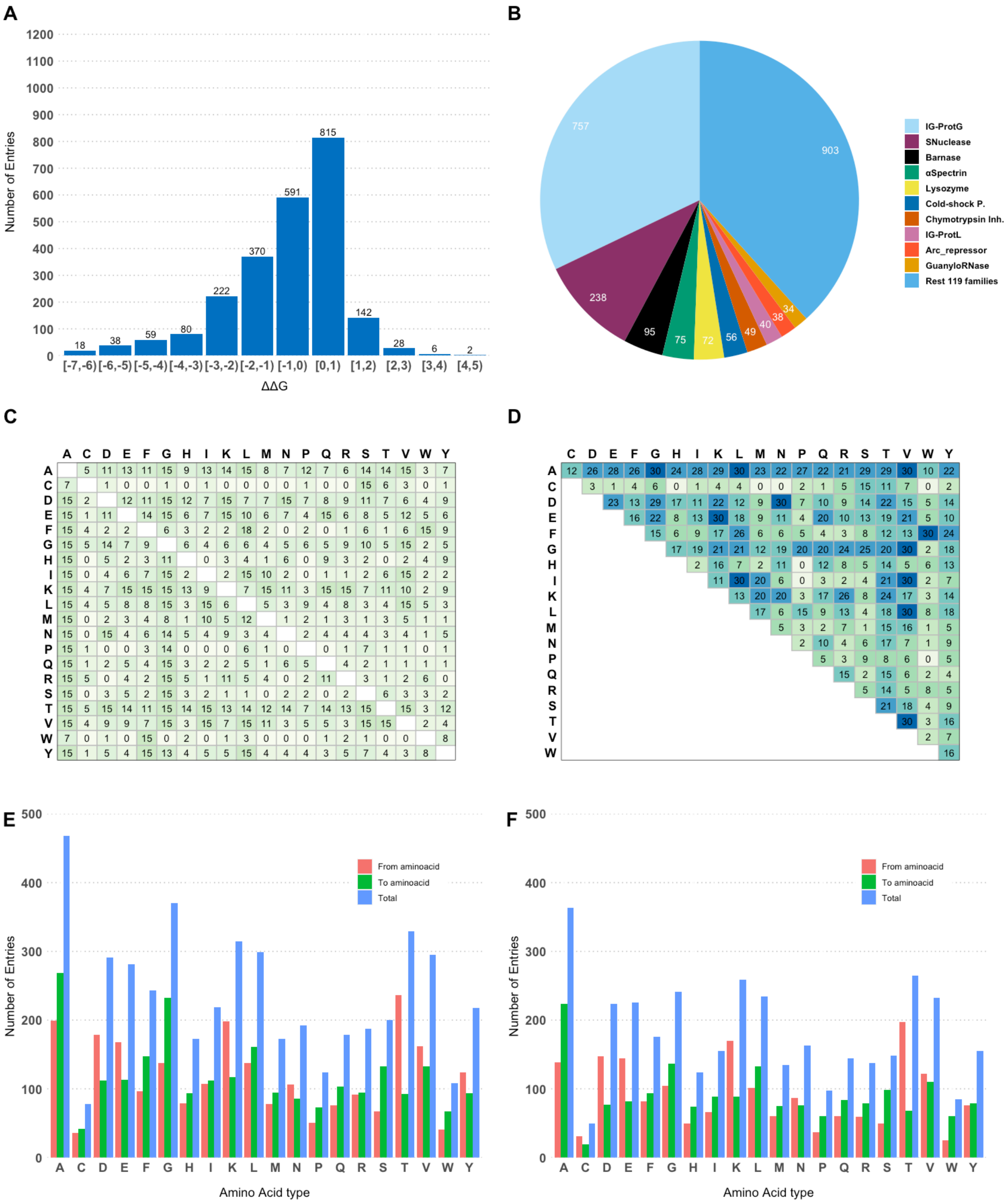

Figure S2. Balanced dataset. The initial curated dataset was restricted to a maximum of 15 test cases per pairwise amino acid mutation to alleviate the alanine over-representation while avoiding to lose stabilizing mutations and protein families. The resulting balanced subset includes 2371 mutations from 129 protein families, 58% destabilizing and 42% stabilizing with an average ΔΔG of -0.7 kcal/mol and a standard deviation of 1.6 Kcal/mol. Panel A-E see Fig S1. Panel F) Number of mutations per amino acid excluding all the cases from the balance subset with a sequence of identity <25% with any of the S^{sym} proteins. This subset no-redundant with S^{sym} includes 1807 mutations from 119 protein families, 52% destabilizing and 48% stabilizing with an average ΔΔG of -0.4 kcal/mol and a standard deviation of 1.3 Kcal/mol.

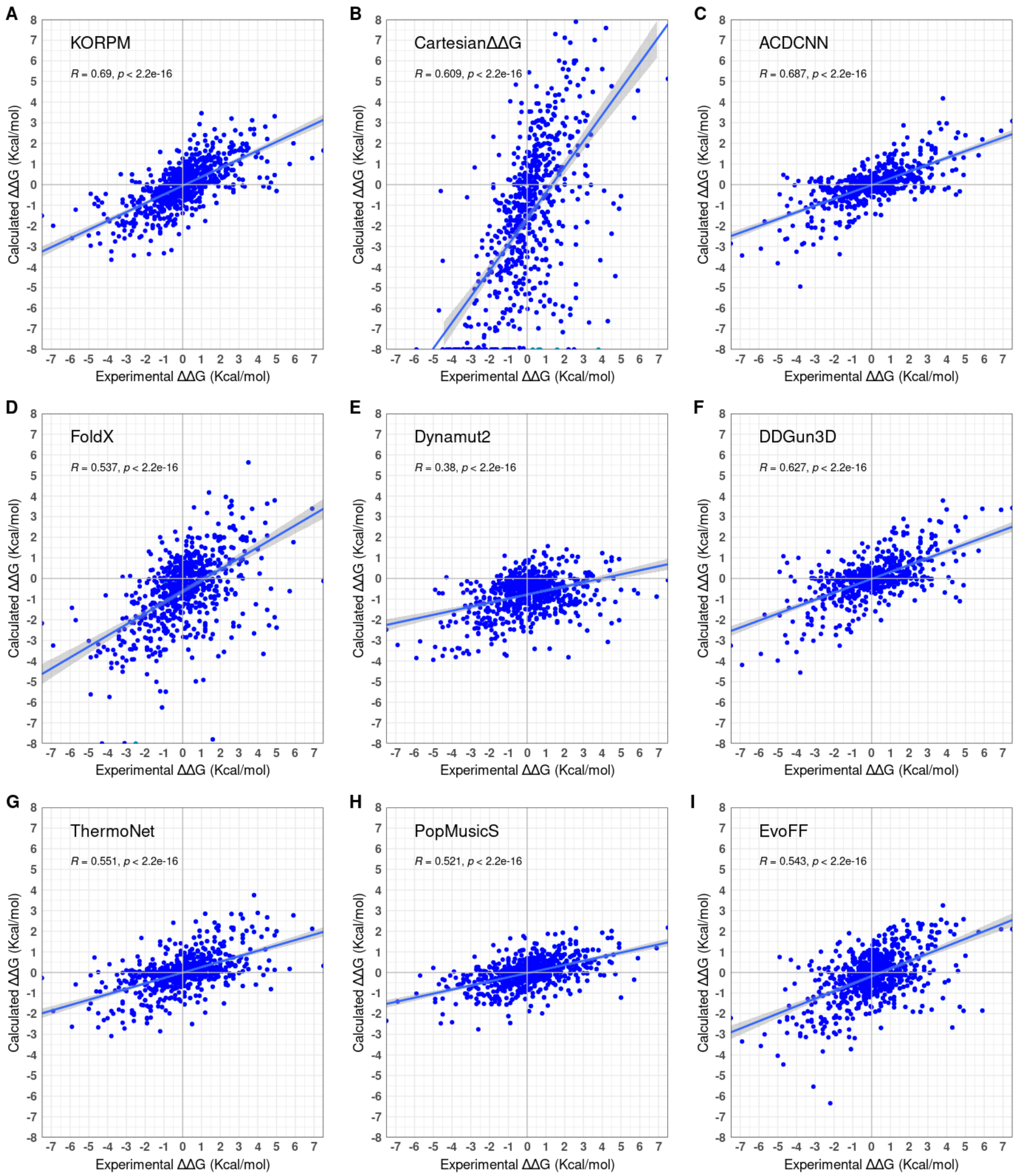

Figure S3. ΔΔG estimations obtained by nine methods tested on S^{sym} dataset.

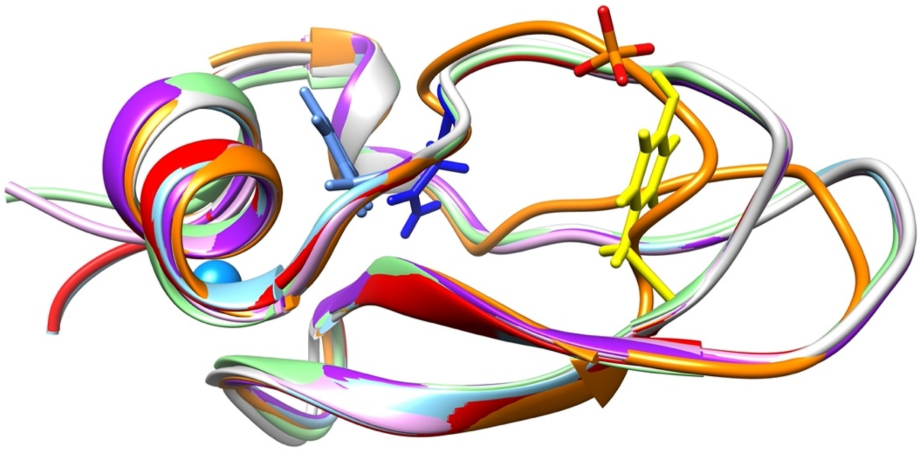

Figure S4. Outlier mutations bovine pancreatic trypsin inhibitor. Mutation Y35G/G35Y (in yellow) corresponds to the largest deviation between experimental and predicted ΔΔG values, 5 vs –3 kcal/mol for the direct mutation and 5 vs -0.4 for the reverse. This corresponds to a large conformational change between the wild-type structure in orange (5PTI) and the mutant (8PTI) in red ribbon. This conformational change is not observed in other bovine pancreatic trypsin inhibitor structures included in the dataset as 1FAN (pink), 1BTI (green), 1BPT (purple), and 1NAG (gray). Notice that in 5PTI structure there is a phosphate group in the vicinity of position 25 (and in other structures) but missing in 8PTI. In this loop region there are two close mutations F45A (light blue) and N43G (dark blue) in where KOPRM underestimates the ΔΔG (*e.g.* direct F45A: -6.9 vs -2.5 and reverse A45F: 6.9 vs 1.6 kcal/mol).

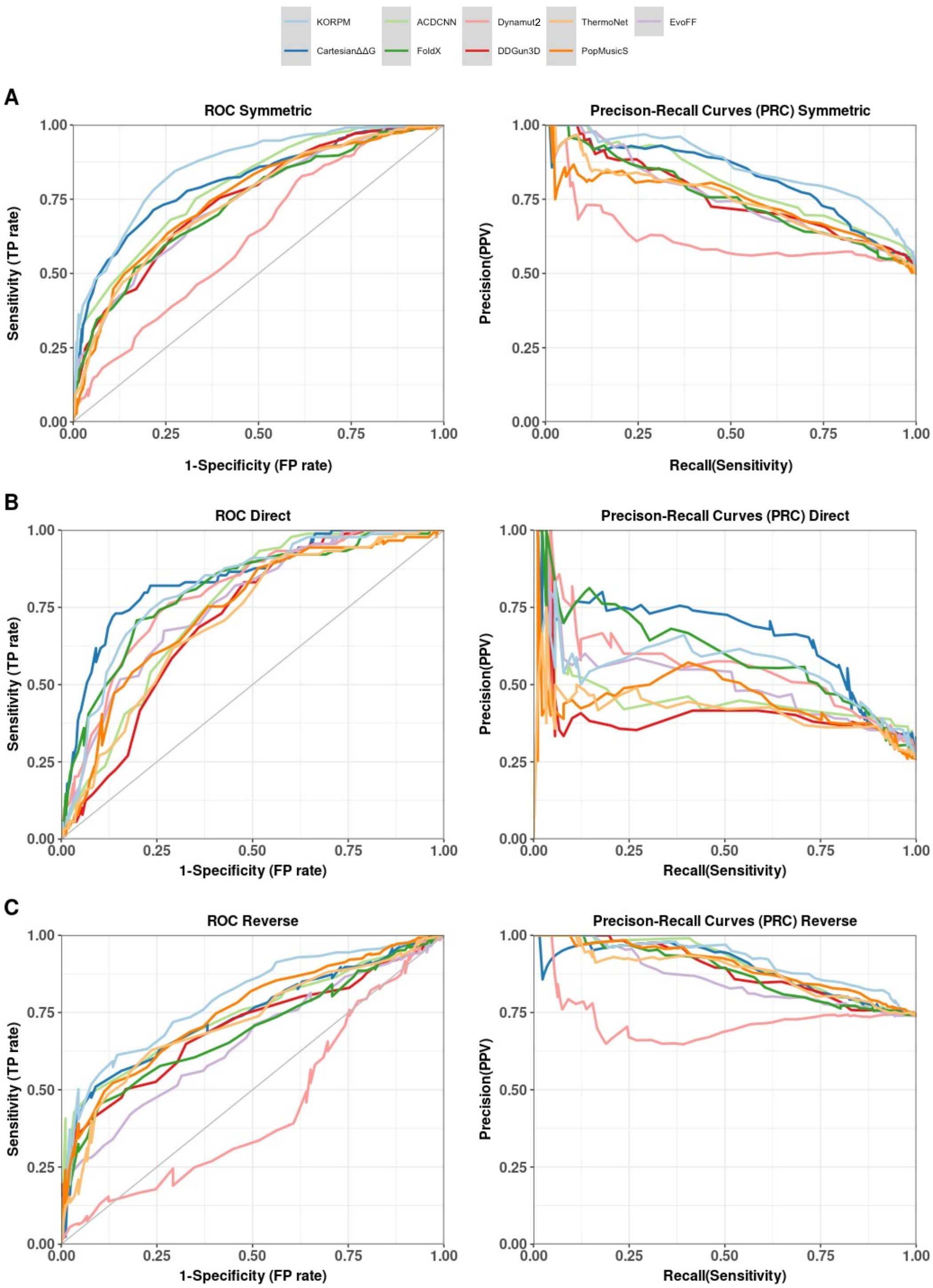

Figure S5. Comparative Receiver Operating Characteristic (ROC) or Precision-Recall (PRC) curves on S^{sym} dataset. A) Results on the whole dataset. B) Results on half direct mutations of the dataset. C) Results on half reverse mutations of the dataset. PRC is more illustrative of the classifier performance with unbalanced datasets (Saito and Rehmsmeier, 2015). KORPM results correspond to the non-redundant Ssym subset (Fig S2, panel F) but practically identical results were obtained with the original balanced subset (Fig 2, panel E).

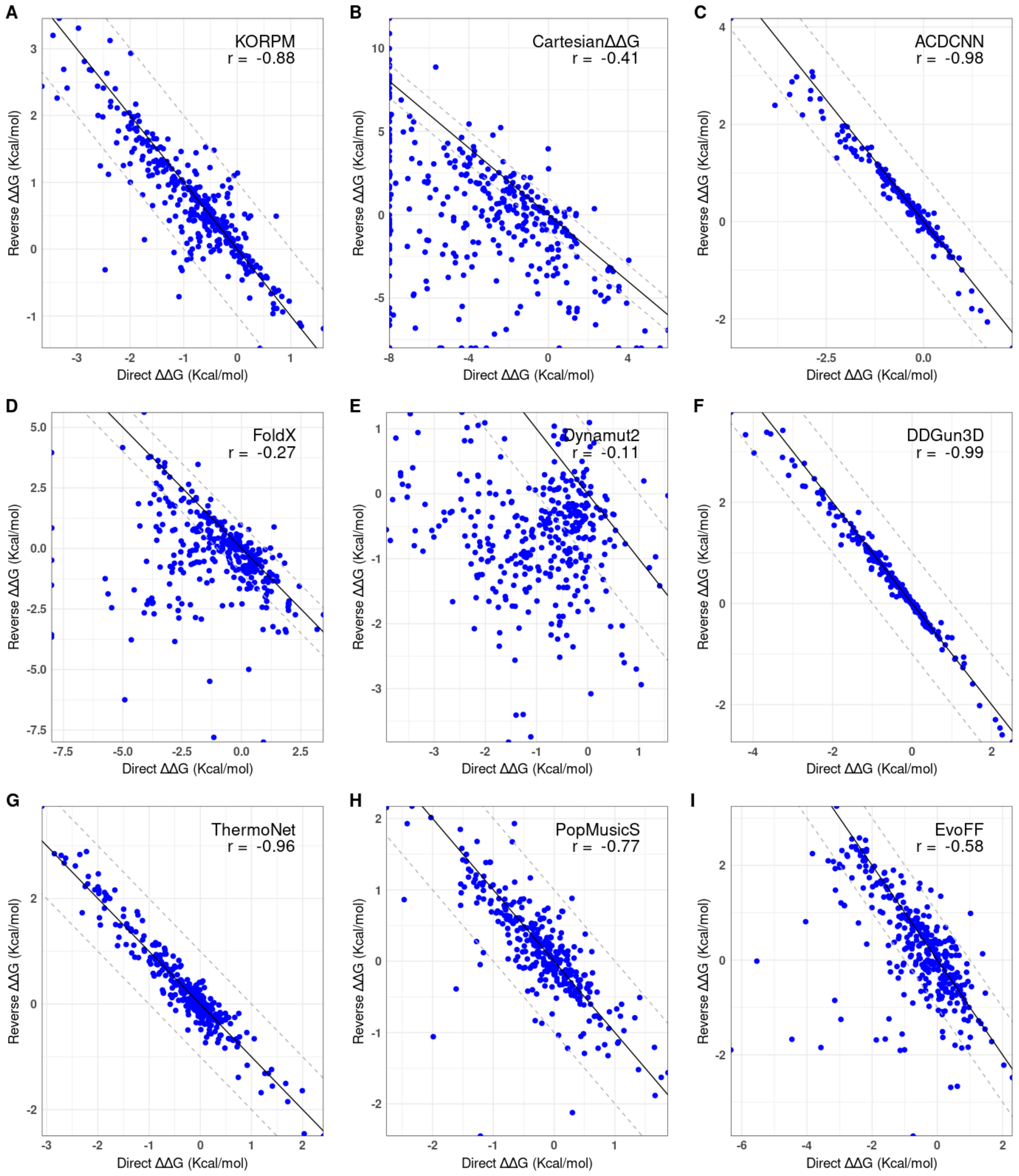

Figure S6. Comparative ΔΔG anti-symmetry results. ΔΔG sum of the predictions for the direct and reverse mutations of the S^{sym} dataset obtained with the tested methods. The ideal relationship $\Delta\Delta G_{direct} + \Delta\Delta G_{reverse} = 0$ is shown as solid line and dashed lines correspond to a variation of ±1 kcal/mol.

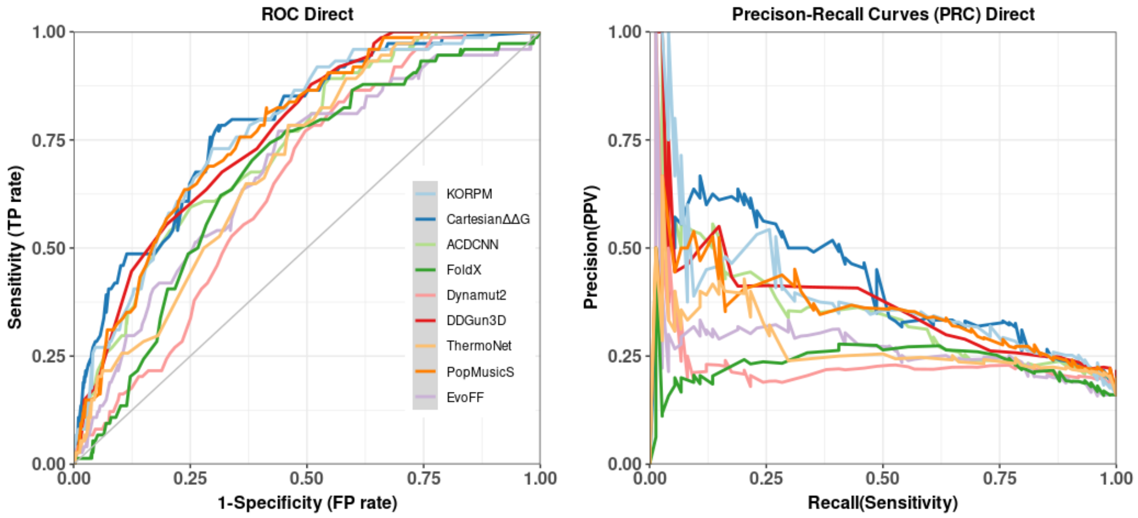

Figure S7. Comparative Receiver Operating Characteristic (ROC) or Precision-Recall (PRC) curves on S461 dataset. PRC is more illustrative of the classifier performance with unbalanced datasets (Saito and Rehmsmeier, 2015). KORPM was trained removing from the balanced dataset any of the S641 proteins with a sequence identity <25%. This non-redundan training subset includes 2224 mutations from 106 protein families, 58% destabilizing and 42% stabilizing with an average ΔΔG of -0.7 kcal/mol and a standard deviation of 1.3 Kcal/mol.

Table S7. Results on direct mutations of S461 dataset (curated from S669).

METHOD	RMSE	MAE	PCC	Sc	Of1	Of2	Sen	Spe	PPV	NPV	ACC	MCC	AUC^{ROC}	AUC^{PRC}
**KORPM**	1.21	0.91	0.57	67.0	32.5	**0.4**	**0.58**	0.79	0.35	**0.91**	0.76	**0.31**	**0.77**	**0.36**
**Cartddg**	3.59	2.93	0.60	58.0	39.6	2.4	0.35	**0.93**	**0.49**	0.88	**0.84**	**0.32**	**0.78**	**0.41**
**FoldX**	1.91	1.26	0.30	65.9	29.3	4.8	0.41	0.79	0.27	0.87	0.73	0.17	0.67	0.23
**EvoFF**	1.28	0.97	0.46	66.4	31.5	2.2	0.45	0.78	0.28	0.88	0.73	0.19	0.68	0.26
**PopMusic-S**	**1.02**	**0.76**	**0.61**	**72.0**	27.5	**0.4**	0.31	**0.92**	**0.43**	0.88	**0.82**	0.26	**0.77**	0.35
**Dynamut2**	1.27	0.96	0.50	64.6	34.1	1.3	0.37	0.74	0.21	0.86	0.68	0.09	0.65	0.22
**DDGun3D**	1.11	0.81	**0.63**	73.1	**25.8**	1.1	0.45	0.88	0.41	**0.89**	0.81	**0.31**	**0.77**	**0.36**
**ThermoNet**	1.24	0.93	0.55	68.3	30.8	0.9	0.50	0.73	0.26	0.88	0.69	0.18	0.70	0.27
**ACDCNN**	**1.07**	**0.78**	**0.61**	**72.7**	**27.1**	**0.2**	0.30	0.89	0.34	0.87	0.79	0.20	0.74	0.34

Appendix I. Corrections of original S^{sym} dataset.

PDB	Mutation	Original	Corrected	Medline References from ThermoMutDB
1BNI	IA96V	-3.1	-0.9	2669964 (-0.90); 1569557 (0.95); 9551101 (-0.80)
1BNI	SA91A	-2.4	-1.8	14516751
1L63	SA44T	0.0	0.01	8289284
1L63	SA38N	0.0	-0.01	1911773
1L63	LA91A	-3.9	-2.6	10545167
1L63	AA130S	1.0	-1.0	8218201
1LZ1	VA2G	-1.3	-2.29	11087397
1LZ1	VA2L	0.3	-0.05	11087397
1LZ1	IA56T	-4.3	-3.6	9010773; 10556244
1LZ1	VA74I	-1.9	0.45	11087397
1LZ1	VA74L	-0.4	0.19	11087397
1LZ1	VA74M	-0.4	0.65	11927576; 11087397
1LZ1	VA74F	0.0	-0.29	11927576;11087397
1LZ1	VA110G	-2.2	0.48	11927576; 11087397
1LZ1	VA110I	-0.8	0.86	11927576; 11087397
1LZ1	VA110F	-1.9	-0.05	11927576; 11087397
2LZM	IA3C	0.0	1.2	3405287
2LZM	RA119E	0.0	-0.04	1942034
4LYZ	GA49A	-0.7	-1.9	11112507; 8771183
4LYZ	GA71A	-2.1	-0.38	11112507
4LYZ	GA102A	-1.2	0.02	11112507
4LYZ	GA117A	-0.8	-1.46	11112507
1RN1	QC25K	1.4	0.93	2663837
2LZM	RA96K	0.0	-0.001	Avoiding zero for the binary classification
1L63	SA44E	0.0	0.001	Avoiding zero for the binary classification
2LZM	KA60P	0.0	-0.001	Avoiding zero for the binary classification

Appendix II. Corrections of S669 dataset.

PDB	#mut	Reference	Notes
2JIE	51	10.1021/acsomega.9b04105	Mutations around the binding site, pdb bound experiment unbound
1XZO	4	10.1021/acs.biochem.7b00833	Mutations at Cu binding site
2VY0	2	10.1111/j.1742-4658.2007.06137.x	Glu53 and Asp287 bind Ca+
1O1U	6	10.1021/bi051781p	Binding pocket, I-BABP with GCA/GCD<br>Identity 25.1% with 1IFC from S2648
4YEE	1	10.1002/cbic.201700589	Carbohydrate binding site.
4YEF	1	10.1002/cbic.201700589	Carbohydrate binding site.
3BCI	2	10.1074/jbc.M707838200	Binding site, unstable disulfide bond
3O39	6	10.7554/eLife.01584	Flexible cradle-shaped dimers, mutants in the binding site
2MPC	3	10.1074/jbc.M114.553305	Binding site, protein-protein interactions
3FIS	1	10.1021/bi050640k	Thermostable dimeric intermediate
2KJ3	9	10.1371/journal.ppat.1004158	β-solenoid prion forming domain, protein-protein interactions
3C2I	11	10.1021/acs.biochem.5b00790	Wrong sign assignment
1FH5	1	10.1016/j.jmb.2016.01.015	Wrong assignment is S21N not S18N<br>0.5-0.6 identity with 2IMM/1LVE from S2648
3D2A	3	10.1002/pro.126	Wrong assignation values are referred to the wt. Thermomut-given mutations (M134E 0.66, M137P 0.98, S163P -0.25) are in the background of 4D3 (thermostable mutant of Bacillus 3D2A which has nine mutations compared to wild-type protein). 3D2A is a thermostable mutant but has three mutations compared with wt.
1FRD	1	10.1002/pro.5560040108	Wrong assignation, in the abstract, said "Strikingly, the Hfd mutant H42R is more stable than the wt Hfd by precisely the amount of stability lost in Vfd upon mutating R42 to H (2.0 kcal/mol)" but uses 4.0 kcal/mol.
3K82	3	10.1371/journal.pone.0098124	Three-state, and four-state models also report misfolding and aggregation
1XWS	4	10.1371/journal.pone.0064824	Unfolding intermediates
1IR3	1	10.1021/bi982546s	Multiple intermediates, in a cleft, also wrong assignation -3.800 not 0.600
3DV0	4	10.1016/j.jmb.2004.12.061	Residues 128 and 129 are missing II130A in the PDB, removed VI129A, VI129G, II130V, II130G
5VP3	3	10.1016/j.saa.2016.01.020	Conformational stability measurements using fluorescence and far-UV CD spectroscopies revealed that all variants unfold in a multi-step manner in which the secondary and tertiary structures are lost in different steps
1PFL	4	10.1515/hsz-2016-0154	Profilin-1 mutants have different structures (Fig 4A), also display aggregation propensity including wt
3S92	1	10.1371/journal.pone.0159180	Different structures wt-mut, see CD in fig 2
4BJX	1	10.1371/journal.pone.0159180	Different structures wt-mut, see CD in fig 2
2CLR	4	10.1021/bi025944a	Cu bind site
1X0J	7	10.1371/journal.pone.0159180	"Most mutations affect significantly protein stability and tertiary structure in solution, suggesting new interactions and an alternative network of protein-protein interconnection as a consequence of single amino acid substitution. (see Fig 3)"

1R6R	4	10.1038/s41598-019-39185-3	Dimer interface, "the mutations significantly affected the secondary structure of the DENV2C protein the mutations L81N and I88N had an even more pronounced impact on the structure of the DENV2C protein, leading to a loss of 58% and 41% in the CD signal"
1A7V	12	10.1016/j.jmb.2009.07.074	His heme loop formation
4N6V	3	10.1110/ps.03270904	form amyloid fibrils "P36G variant is most prone to amorphous aggregation", P79S variant is a dimer
1PRG	7	10.3390/ijms18020361	"non-two-state dependence upon increasing urea concentration for the wild type and for the variants Q286P, R288H, V290M, R357A, F360L, and P467L (Figure 5B)"
1MN1	2	10.1073/pnas.96.20.11247	they use a complex of Actin with a domain of Gelsolin and make the mutation in the Actin chain. But the experimental data is for mutations in another domain of Gelsolin
2PR5	14	10.1371/journal.pcbi.1003129	is a dimer, H22, V25, N107, D109, M111, V120 and N124 are from the dimer interface, also bound to FMN
1GWY	1	10.1016/j.abb.2017.11.005	Binding to DOPC/SM/Chol (1:1:1) vesicles studied by ITC, with lipids forming pores
2KS4	2	10.1016/j.abb.2017.11.005	Binding to DOPC/SM/Chol (1:1:1) vesicles studied by ITC, with lipids forming pores, also wrong sign assignment
1GLU	1	10.1016/j.abb.2017.11.005	Zinc binding site, dimer, DNA bound
1HCQ	2	10.1016/S0022-2836(02)00236-X	Zinc binding site, dimer, DNA bound
1N18	4	10.1073/pnas.0913021108	HIS 46 binds Cu, PDB is a dimer. Wt is a monomer, mutants tend to aggregate. In the text "Attempts to fit the data to two-state models for monomer/dimer, monomer/trimer, and monomer/tetramer transitions gave poor fits with nonrandom residuals, indicating that the association is likely more complex than a simple two-state process"
3ECU	1	10.1073/pnas.0913021108	Wt is a monomer, mutants tend to aggregate.
1PRE	1	10.1074/jbc.274.51.36722	Heptameric, unfold in two steps
1SPD	2	2254318	Superoxide dismutase is a dimer
2JUC	3	10.1016/j.redox.2017.10.022	mutations forms β-sheet-rich amyloid fibrils
2DVV	2	10.1021/ja076628s	Most mutations affect significantly protein stability and tertiary structure in solution, suggesting new interactions and an alternative network of protein-protein interconnection as a consequence of single amino acid substitution. see Fig 3,
2OUO	1	10.1371/journal.pone.0159180	Most mutations affect significantly protein stability and tertiary structure in solution, suggesting new interactions and an alternative network of protein-protein interconnection as a consequence of single amino acid substitution. see Fig 3,
1OSI	4	10.1093/protein/14.8.601	is a dimer, the structure corresponds to a thermostable A172L mutant
1F8I	1	10.1038/s41598-017-01235-z	is a tetramer
1H0X	2	10.1007/s00792-009-0233-7	Wrong assignation, it is a dimer, a rare case in which experimentally demonstrates that mutations did not affect the dimer structure. However, ddG values must be divided by 2.0 because predictions were done with the monomer.
1D5G	2	10.1016/j.jmb.2006.08.076	Ligand binding site, PDZ2 bound to RA-GEF2 peptide
4WAA	3	10.1038/s41598-017-01258-6	Binding values, the title of the table where extracted said "Thermodynamic parameters of interaction between LC3B (wild type and mutants) and Nix"
2RPN	2	10.1371/journal.pone.0146232	43 identity with 2A36 from S2648
3G1G	4	10.1002/prot.24188	25 identity with 1A43 from S2648
2BJD	3	https://doi.org/10.1021/ja076628s	31 identity with 1APS from S2648
